# Transactions of the Ninth International Medical Congress

**Published:** 1888-08

**Authors:** 


TRANSACTIONS OF THE NINTH INTERNATIONAL MEDICAL CONGRESS.—Two volumes of the Transactions of the recent Medical Congress in Washington, are ready and being distributed to members as rapidly as possible. Through the active agency of the late Secretary-General Hamilton, one hundred and fifty members received these volumes while in attendance on the annual meeting of the American Medical Association, in Cincinnati. The work of publication has been done in excellent style thus far. The third volume is also by this time complete, and the remaining two will follow within the next three months; making five large volumes, and constituting a valuable library in themselves.

